# Systematic Review for the Medical Applications of Meditation in Randomized Controlled Trials

**DOI:** 10.3390/ijerph19031244

**Published:** 2022-01-22

**Authors:** Do-Young Kim, Soo-Hwa Hong, Seung-Hyeon Jang, So-Hyeon Park, Jung-Hee Noh, Jung-Mi Seok, Hyun-Jeong Jo, Chang-Gue Son, Eun-Jung Lee

**Affiliations:** 1 Korean Medical College, Daejeon University, 62, Daehak-ro, Dong-gu, Daejeon 34520, Korea; 95kent@naver.com (D.-Y.K.); 1052akdp@naver.com (S.-H.H.); jshjhi@naver.com (S.-H.J.); jenpsh@gmail.com (S.-H.P.); dhy03065@naver.com (J.-H.N.); cococ000@naver.com (J.-M.S.); perarduaadastra9@naver.com (H.-J.J.); 2 Department of Liver and Immunology Research Center, Daejeon Oriental Hospital of Daejeon University, 75, Daedeok-daero 176, Seo-gu, Daejeon 35235, Korea; 3 Department of Korean Rehabilitation Medicine, Daejeon Oriental Hospital of Daejeon University, 75, Daedeok-daero 176, Seo-gu, Daejeon 35235, Korea

**Keywords:** meditation, RCT, review, depression, sleep, fatigue

## Abstract

Background: Meditation has been increasingly adapted for healthy populations and participants with diseases. Its beneficial effects are still challenging to determine due to the heterogeneity and methodological obstacles regarding medical applications. This study aimed to integrate the features of therapeutic meditation in randomized controlled trials (RCTs). Methods: We conducted a systematic review of RCTs with meditation for populations with diseases using the PubMed database through June 2021. We analyzed the characteristics of the diseases/disorders, participants, measurements, and their overall benefits. Results: Among a total of 4855 references, 104 RCTs were determined and mainly applied mindfulness-based (51 RCTs), yoga-based (32 RCTs), and transcendental meditation (14 RCTs) to 10,139 patient-participants. These RCTs were conducted for participants with a total of 45 kinds of disorders; the most frequent being cancer, followed by musculoskeletal and connective tissue diseases and affective mood disorder. Seven symptoms or signs were frequently assessed: depressive mood, feeling anxious, quality of life, stress, sleep, pain, and fatigue. The RCTs showed a higher ratio of positive outcomes for sleep (73.9%) and fatigue (68.4%). Conclusions: This systematic review produced the comprehensive features of RCTs for therapeutic meditation. These results will help physicians and researchers further study clinical adaptations in the future as reference data.

## 1. Introduction

Meditation is a mental practice aiming to improve the psychological capacity of self-regulation regarding attention, awareness, and emotion [1]. Its implementation has been described not only in religious and cultural beliefs but also in health promotion as itself or as a component of mind–body practices such as yoga, qigong, tai chi, and mindfulness-based interventions (MBIs) [2]. The US National Center for Complementary and Integrative Health (NCCIH) reported the health benefits of meditation, including the regulation of blood pressure and blood glucose and stress reduction for healthy participants [3]. From 2012 to 2017, the use of meditation more than tripled from 4.1% to 14.2% in the general US adult population [4].

Additionally, meditation for patients in clinics against diverse disease conditions, such as complementary and alternative medicine (CAM), has been demanded [5]. Therapeutic meditation techniques include MBIs, yoga-based programs, and transcendental meditation (TM) [2]. Mindfulness-based stress reduction (MBSR) and mindfulness-based cognitive therapy (MBCT), which are representative MBIs, have presented psychological benefits for patients with depression and cancers [6,7]. Yogic meditation and TM also produce preventive benefits regarding risk factors for cardiovascular diseases and affective disorders by regulating brain activity and the autonomic nervous system [8,9].

However, it is difficult to objectify the effect of meditation because of its methodological obstacles, such as diverse task forms, a wide range of traditional roots, and the proficiency of practicians [10]. The NCCIH pointed out that scientific research on meditation practices has a heterogeneous theoretical perspective and poor study design quality [3]. Most of the 400 clinical studies for meditation conducted before 2005 were considered to be poorly designed [11], while more recent meditation-derived clinical studies include many well-designed trials [12,13]. Physicians need to consider the characteristics of meditative intervention and its expected effect for application in their treatment plans. To date, no systematic analysis showing the current status of clinical applications for patients exists.

To facilitate these works, this study comprehensively reviewed the features of randomized controlled trials (RCTs) with meditative interventions designed for patients to date regarding patients, interventions, controls, measurements, and overall results.

## 2. Methods

### 2.1. Database and Searching Strategy

The literature survey of this review was conducted in accordance with the Preferred Reporting Items for Systematic Reviews and Meta-Analysis (PRISMA) guidelines [14] using the PubMed electronic database through 10 June 2021. The search term used was ‘meditation [Title/Abstract]’ with excluding filters [Systematic review, Review, Meta-analysis]. The literature survey was conducted by 6 individual reviewers, and the third one checked the process.

### 2.2. Eligibility Criteria

The literature screening process of this study used the following inclusion criteria: (1) studies that were RCTs or randomized controlled crossover trials; (2) the study participants were patients with diseases; and (3) studies that evaluated the therapeutic efficacy of meditation. The exclusion criteria were as follows: (1) articles with no full text; (2) pilot RCTs or post-analysis studies; (3) studies with a Jadad score less than 3 points; and (4) studies with results published in languages other than English.

### 2.3. Data Extraction and Quality Assessment

Data extraction dealt with the number of participants, mean age, subjects’ diseases, intervention category, treatment period, control, and outcome measurements. We also obtained the result data of the original article with a statistical analysis of the treatment effect compared to the control.

To assess the quality of the RCTs, the Jadad scale was used [15]. The Jadad scale is a five-point checklist scale that allocates points for descriptions of randomization, double-blinding, withdrawals, and drop-outs. Trials with ≥3 points were considered high quality and were included in this study.

### 2.4. Categorization of Meditation

We categorized meditative interventions into 3 groups (Mindfulness- and yoga-based meditation and TM). Mindfulness-based or yoga-based meditation included interventions with the terms “mindfulness” or “yoga” used in each original article. The TM group included TM, mantras, and spiritual meditation.

### 2.5. Judgment of the Statistical Efficiency of the Intervention

We judged the benefit of intervention with statistical significance based on the data presentations of the original articles. In general, ‘statistical significance’ meant that the intervention showed statistically significant improvement (intervention vs. control, *p* < 0.05 or Cohen’s d > 0.5) according to the outcome measurement at the planned time point or the closest time to the end of the treatment period. We defined ‘benefit with statistical significance’ for the following cases: (1) One or more of the outcomes regarding target symptoms or signs were statistically significant, or (2) statistical significance was observed only at the planned time point or the closest point to the period after treatment, unless any description was noted.

### 2.6. Data Analysis

This systematic review did not need to apply statistical analysis. Regarding the number of participants, age, and treatment periods in the demographic features, the data are presented as the mean and standard deviation (SD) computed by Microsoft Excel software.

## 3. Results

### 3.1. General Characteristics of the RCTs

**Figure 1:**
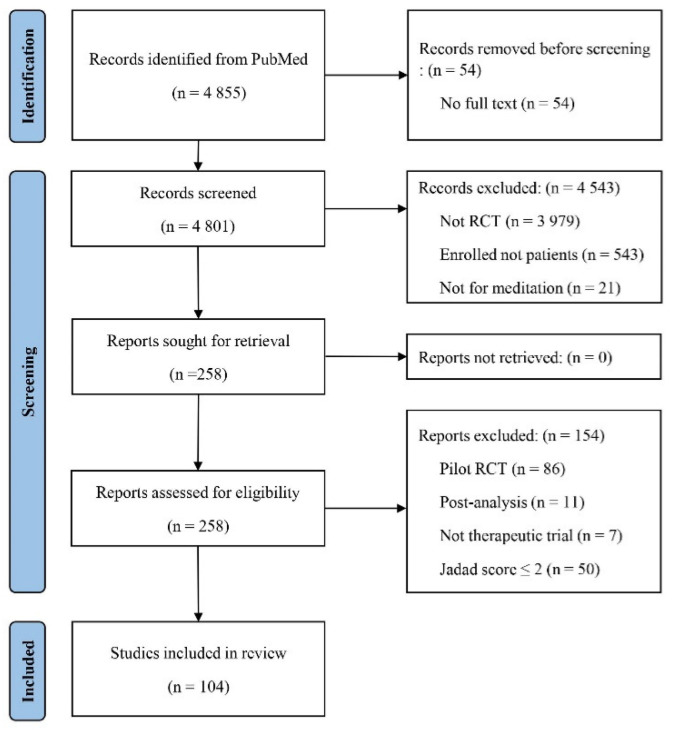
Flow chart of the study.

A total of 4855 initial references were identified from PubMed, and 104 articles met the inclusion criteria of this review (Figure 1). In the 104 RCTs, 10,139 (3117 males and 7022 females) patients participated (mean age: 47.6 ± 13.1 years), and the mean treatment period was 10.3 ± 9.1 weeks (Table 1). The controls included usual therapy (29 RCTs), waitlist (16 RCTs), education (15 RCTs), and relaxation (11 RCTs) (Table 2 and Table 3). The RCTs were predominantly conducted in the USA (*n* = 41), followed by India (*n* = 15) and Germany (*n* = 9) (data not shown).

### 3.2. Diseases of the Participants and Types of Meditation in the RCTs

Among a total of 104 RCTs, 73 trials (70.2%) were performed for participants with physical diseases, and 31 (29.8%) were performed for participants with mental disorders (Table 2 and Table 3). The physical diseases included cancer (16 RCTs), followed by musculoskeletal and connective tissue (14 RCTs), nervous system (9 RCTs), circulatory system and gynecological diseases (7 RCTs each). On the other hand, affective disorder (13 RCTs) and post-traumatic stress disorder (PTSD, 7 RCTs) were the most frequent mental diseases in the subjects (Table 1).

In terms of the interventions used in the RCTs, 34 kinds of meditations were employed. Ninety-three trials (89.4%, out of 104 total) employed at least one of the three major meditative interventions: mindfulness-based (51 RCTs), yoga-based (32 RCTs), or TM (14 RCTs) (Table 1). Further detailed information for the diseases/disorders and types of meditations is shown in Table 2.

### 3.3. Target Measurements in the RCTs

**Figure 2:**
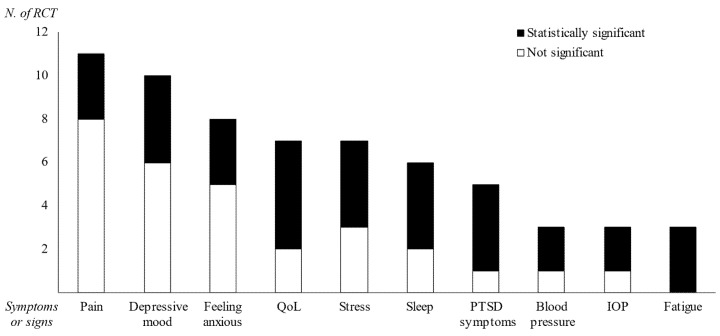
**Graphical display of the primary outcomes of the RCTs.** Regarding only primary measurements, the 10 most frequently assessed symptoms or signs (conducted in ≥3 RCTs) are shown. The black square (■) indicates the number of RCTs in which the intervention achieved statistical significance compared to the control (*p* < 0.05 or Cohen’s d > 0.5) for the primary outcome assessment.

From a total of 104 RCTs, the mean number of assessed outcomes per RCT was 3.9 ± 2.5, which presented 105 kinds of outcomes (Table 1). A total of 76 RCTs (73.1%) reported primary outcome measurements, which produced 99 primary outcomes for 41 kinds of measurements. Ten kinds of measurements were reported in at least three RCTs, including pain (11 RCTs), depressive mood (10 RCTs), feeling anxious (8 RCTs), quality of life (QoL, 7 RCTs), stress (7 RCTs), sleep (6 RCTs), PTSD symptoms (5 RCTs), blood pressure, intraocular pressure (IOP), and fatigue (3 RCTs each) (Figure 2).

In the sum of all measurements (406 measurements in 104 RCTs), depressive mood symptoms were most frequent (56 RCTs, 53.8%), followed by feeling anxious (40 RCTs, 38.5%), QoL (32 RCTs, 30.8%), stress (31 RCTs, 29.8%), sleep (23 RCTs, 22.1%), pain (22 RCTs, 21.2%) and fatigue (19 RCTs, 18.3%) (Table 1).

### 3.4. Clinical Outcomes for the Primary Measurement and Total Measurements

From a total of 99 primary outcomes (41 kinds of measurements), the top 3 most frequent outcomes were pain, depressive mood, and feeling anxious, and they did not reach a 50% positive ratio (27.3%, 40.0%, and 37.5%, respectively). QoL (71.4%), stress (57.1%), sleep (66.7%), PTSD symptoms (80.0%), blood pressure (66.7%), IOP (66.7%), and fatigue (100.0%) showed over half the ratio of positive results (Figure 2).

**Figure 3:**
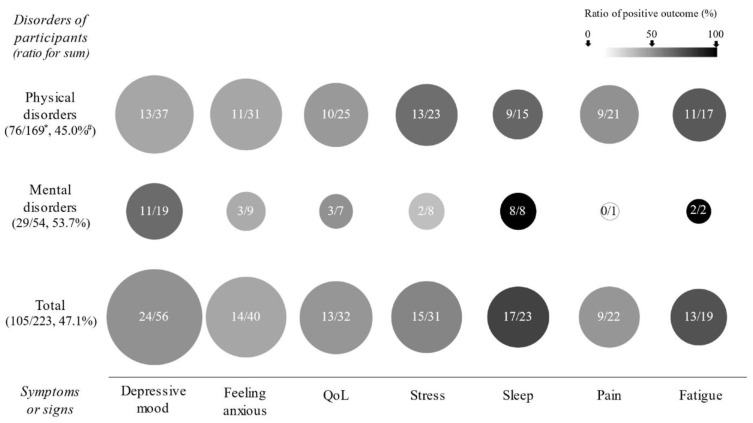
**The features of significant outcomes in the RCTs according to physical and mental disorders.** Regarding all measurements, the seven most frequently assessed outcomes are displayed according to subjects with physical and mental disorders. The circle’s size and level of darkness indicate the number of RCTs and the ratio of positive outcomes, respectively. The positive outcome indicates that the treatment achieved statistical significance compared to the control (*p* < 0.05 or Cohen’s d > 0.5). */# indicates the total number of outcomes measured for the seven symptoms in the RCTs for patients with given disorders and positive outcomes and their ratios.

As shown in Figure 3, RCTs for sleep and fatigue showed 100% positive effects in RCTs with participants with mental disorders and showed a relatively high ratio of positive outcomes in all the RCTs (73.9% and 68.4%, respectively), followed by stress (48.4%), depressive mood (42.9%), pain (40.9%), QoL (40.6%), and feeling anxious (35.0%). The positive ratios of depressive mood and stress symptoms were greater than 50% only for mental and physical diseases (57.9 and 56.5% each) (Figure 3).

### 3.5. Clinical Outcomes According to the Type of Meditation

**Figure 4:**
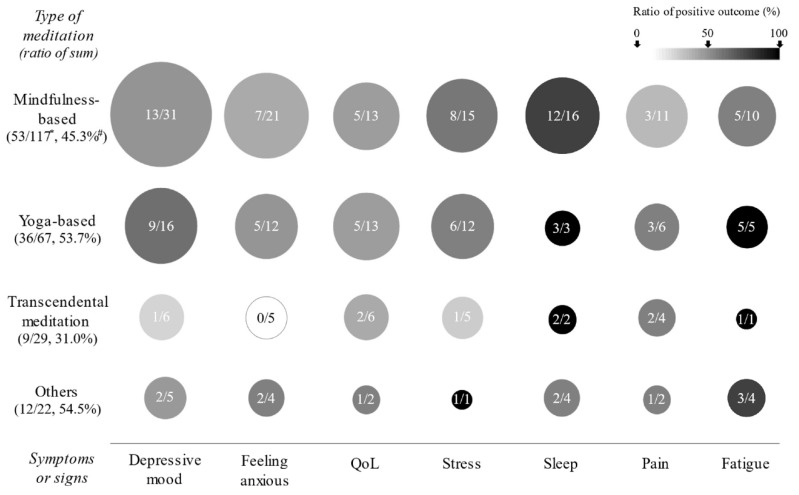
**The features of significant outcomes in the RCTs according to the types of meditations.** Regarding all measurements, the seven most frequently assessed outcomes are displayed according to the types of meditations. The circle’s size and level of darkness indicate the number of RCTs and the ratio of positive outcomes, respectively. The positive outcome indicates that treatment achieved statistical significance compared to the control (*p* < 0.05 or Cohen’s d > 0.5). */# indicates the total number of outcomes measured for the seven symptoms in the RCTs for patients with given disorders and positive outcomes, and their ratio.

Among the three major meditative interventions, yoga-based interventions (53.7% of 67 outcomes) showed slightly higher positive outcomes than mindfulness-based interventions (45.3% of 113 outcomes) and TM interventions (31.0% of 29 outcomes). Compared to other symptoms, sleep-targeted RCTs most frequently employed mindfulness-based meditation (16 of 25 RCTs), which presented over half the ratio of positive results (75.0%). Yoga-based interventions showed higher positive results regarding depressive mood (56.3%). RCTs for sleep and fatigue showed 100% of significant benefits by mindfulness-based meditation and TM (Figure 4).

## 4. Discussion

Research on the physiological effects of meditation was first conducted in the 1950s, and a clinical study was initiated in the 1970s [118]. While CAM is becoming increasingly popular, so does the employment of meditation for disease therapy currently representing a quarter of the total CAM uses [119]. Although numerous studies have been conducted to investigate the clinical benefits of meditation, the standardization of its benefits is still challenging because of the heterogeneity and methodological weaknesses [11]. To provide fundamental information for future clinical use and study, this review aimed to produce features of clinical applications with meditation using RCTs for especially diseased populations to date.

The trials included in this study gradually increased from 3 RCTs over a 5 year period from 2000 to 2004 to 52 RCTs in the period from 2015 to 2019 (data not shown). The sex ratio of participants showed an approximately twofold higher distribution in females (7022 out of a total of 10,139), generally correlating with sex differences in the prevalence of meditation use [120]. The 104 RCTs included in this study involved 45 kinds of disease conditions, and 43 RCTs assessed the primary outcomes directly related to the target diseases/disorders, such as pain for arthritis [33,44], sleep quality for sleep disturbance [114], and the PTSD-symptom scale for people living with PTSD [13,101,102,103]. Several RCTs evaluated objective changes by assessing blood markers, heart rate variability [12,32,59,61,72,74,76,77,92,93,103], or brain imaging scans [53], as summarized in Table 2 and Table 3. The others mainly focused on disease-related comorbid symptoms, including depressive mood, feeling anxious, QoL, and stress (Figure 2).

The top 7 most frequently assessed clinical outcomes included depressive mood, feeling anxious, QoL, stress, sleep, pain, and fatigue, and at least 1 of them was measured in 87.5% (91 RCTs) of the 104 RCTs. These symptoms have a high prevalence in both diseased and healthy populations. For example, US survey data presented prevalence rates of 21%, 25%, and 33% for depressive mood, fatigue, and pain, respectively, in subjects with a cancer history and 18%, 18%, and 29% in adults without a cancer history, respectively [121]. Patients usually complained of clusters of several symptoms simultaneously; accordingly, most RCTs had multiple outcomes, with an average of 3.9 ± 2.5 measurements (Table 1). Mind–body interventions such as relaxation, cognitive behavioral therapy, coping skills training, or meditation helped patients manage symptom clusters of pain–fatigue–sleep disturbance in cancer patients [122].

Meditative interventions are usually expected to promote strengths in tolerating individual torments by reinforcing the psychological capacity of self-regulation [123]. In our data, meditations most frequently targeted depressive mood and feeling anxious, which were known as the most common comorbid symptoms in the population under disease conditions [124,125]. Both complaints were significantly improved by meditation practices by 42.9% and 35.0%, respectively, and the anti-depressive effect was more prominent in patients with mental disorders (57.9%) than in those with physical disorders (35.1%) (Figure 3). The therapeutic mechanisms of meditation against major depressive disorders are understood as upregulation of serotonin and dopamine along with neuro-immuno-endocrinological modulation [126]. Sleep quality and fatigue-related benefits were the most positive in the current study by 73.9% and 68.4%, respectively, which increased further in subjects with mental disorders (100.0% both) (Figure 3). In disease conditions, sleep-requiring symptoms such as fatigue and lethargy are common complaints as pathophysiological defense responses [127]. The role of meditation in sleep quality is understood as changing sleep architecture by regulating brain activities [128]. Accumulated evidence indicates that meditative interventions could lead to functional and structural changes within the brain, such as the prefrontal region, cingulate cortex, striatum, and amygdala, suggesting its promising therapeutic effect on mental disorders [129].

In terms of QoL and stress symptoms, they are the main reasons for the use of meditation by meditation practitioners in the US [120]. The benefits of mindfulness- and yoga-based programs for stress reduction and global wellness in nonclinical populations are widely and well-known [130,131]. However, our data suggest that meditative interventions did not consistently improve QoL or stress symptoms in disease conditions. Perhaps disease-related QoL and stress differ from those of healthy conditions due to the intensity of root matters such as histological and functional impairments [132]. Several RCTs on gynecological disease [62,64,67] and PTSD [103,104], however, showed fragmentary evidence to support the stress reduction effect without ineffective outcomes (Table 2 and Table 3).

In general, RCTs for physical disease included more than two mental disorders (74 physical vs. 31 mental diseases). Among them, cancer, musculoskeletal or connective tissue, and nervous system diseases were dominant (16, 14, and 9 RCTs, respectively) (Table 1). For patients with cancer, meditation is one of the preferred CAMs with the highest satisfaction [133]. In our data, the interventions showed a decent ratio of positive results (positive, 8, vs. not significant, 2) in terms of cancer-related fatigue (CRF) (Table 2). Regarding the pain relief effect, the most of RCTs evaluating pain were for diseases of the musculoskeletal, connective tissue, and nervous system (13 RCTs out of 22). Although they failed to present consistent benefits (7 positive vs. 6 not significant), this ratio was higher than that in other diseases (2 vs. 6) (Figure 3, Table 2 and Table 3).

Overall, according to our data, meditative therapies appear to be more beneficial for improving sleep quality and fatigue management as well as anti-depressive effects, especially in patients suffering from mental disorders. The conceptual basis of meditations includes attention, awareness, point of focus, or self-transcendence, even though they vary depending on historical and regional beliefs and methodological heterogenicity [134]. When we compared the gross features of three major meditation classifications, approximately 50% of the RCTs (51 RCTs) employed mindfulness-based meditation (Table 1). Compared to other symptoms, sleep-targeted RCTs (64%) mainly employed mindfulness-based meditation, resulting in relatively high positive results (75.0%). The second most frequently applied type of intervention was yoga-based interventions, which were more beneficial for depressive mood (56.3%) than other interventions (Figure 4). In general, physical exercise therapies are recommended for patients suffering from most diseases, including cancer, fibromyalgia, and multiple sclerosis [135,136,137]. However, they could often worsen certain symptoms, such as fatigue, or occasionally cause other physical adverse effects [138]. Some studies have shown that managing concurrent symptoms well has a medical impact, including on the final clinical outcome; for example, reducing fatigue severity affects the survival or recurrence rate in breast cancer patients [139]. In this regard, meditation could be a promising alternative to manage subjective complaints in people living with various diseases.

This review has several limitations. First, a single database (PubMed) was used for the literature survey. Although the majority of data were extracted from the PubMed database, more evidence could be provided by other databases. We also found that the generally poor design quality of the studies remains a weakness. In this review, the number of articles were reduced by two-thirds in the screening process with Jadad scores (Figure 1). Further rigorously designed clinical trials are needed to objectify the clinical benefits of meditation. To provide confident information, pilot RCTs were excluded from this review. They commonly enrolled a small number of participants, focusing mainly on the feasibility of further trials. However, this tactic also has a risk of losing any valuable data.

Despite the limitations mentioned above, this review produced the comprehensive features regarding patients, interventions, controls, measurements, and their overall benefits in diseased populations. These results provide fundamental information to patients, practitioners, and researchers as reference data for future studies.

## Figures and Tables

**Table 1:** Study characteristics.

Items	Count
N. of RCT	104
N. of participants (female)	10,139 (7022)
Mean N. of participant (the number ± SD)	97.3 ± 71.4
Mean age (year ± SD) ^A^	47.6 ± 13.1
Mean treatment period (week ± SD)	10.3 ± 9.1
Disorders of the participants (N. of RCT, %)	104 (100)
Cancer	16 (15.4)
Diseases of the musculoskeletal and connective tissues	14 (13.5)
Affective mood disorder	13 (12.5)
Disease of the nervous system	9 (8.7)
Disease of the circulatory system	7 (6.7)
Gynecological disorder	7 (6.7)
Post-traumatic stress disorder (PTSD)	7 (6.7)
Metabolic disorder	6 (5.8)
Disease of otorhinolaryngology	5 (4.8)
Others	20 (19.2)
Type of meditation ^B^ (N. of RCTs, %)	
Mindfulness-based meditation	51 (49.0)
Yoga-based meditation	32 (30.8)
Transcendental meditation	14 (13.5)
Others	11 (10.6)
Main clinical outcomes ^B, C^ (N. of RCTs, %)	
Mean N. of measurements per RCT (the number ± SD)	3.9 ± 2.5
Depressive mood	56 (53.8)
Feeling anxious	40 (38.5)
Quality of life (QoL)	32 (30.8)
Stress	31 (29.8)
Sleep	23 (22.1)
Pain	22 (21.2)
Fatigue	19 (18.3)

^A^ This is the mean of the participants’ ages presented as the median or mean in the original articles. ^B^ These items have been applied multiple times, thus the sum of percentages is larger than 100%. ^C^ These items shown in the table are the most frequently used in RCTs.

**Table 2:** Summary of the RCTs for participants with physical disorders.

Disorder [Reference]	Kind of Meditation (Category *)	N. of Participants ^#^ (N. of Arms, Controls)	Period (Weeks)	Clinical Finding (Statistical)	
				Significant	Not Significant
Cancer					
Breast cancer [16]	Kirtan Kriya (Y + T)	31 (2, music listening)	8	depressive mood, fatigue, cognitive functions ^A^,	feeling anxious, stress, self-efficacy
Breast cancer [17]	MM (M)	92 (3, muscle relaxation, education)	12	fatigue ^A^	QoL
Breast cancer [18]	Self-care toolkit (other)	100 (2, usual care)	2	pain	depressive mood, feeling anxious ^A^, sleep, fatigue, social role, physical functions, nausea
Breast cancer [19]	Tai chi (other)	90 (2, CBT)	12	depressive mood, sleep ^A^, fatigue	-
Breast cancer [20]	Yoga (Y)	69 (2, counseling)	24	depressive mood, feeling anxious, cancer symptoms	-
Breast cancer [21]	Hatha yoga (Y)	40 (2, waitlist)	12	QoL, fatigue, menopausal symptoms ^A^	depressive mood, feeling anxious
Breast cancer [22]	YOCAS (Y)	167 (2, waitlist)	4	fatigue	-
Breast cancer [23]	Qigong + Tai chi (other)	87 (2, sham qigong)	12	fatigue ^A^	depressive mood, sleep
Breast cancer [24]	Mindful awareness practices (M)	71 (2, usual care)	6	stress ^A^, sleep, fatigue, hot flashes, affect	depressive mood ^A^, pain, fear, intrusive thoughts
Breast cancer [25]	Mindfulness-based cancer recovery (M)	271 (3, supportive therapy, stress management)	8	stress, mood ^A^	health-related functions, social support
Cancer [26]	Yoga (Y)	410 (2, usual care)	4	sleep ^A^	-
Breast cancer [27]	MBSR (M)	336 (2, usual care)	8	-	sleep ^A^
Breast cancer [28]	Danhak (other)	102 (2, usual care)	6	feeling anxious ^A^, fatigue, QoL	depressive mood ^A^, dyspnea
Cancer [29]	MBSR (M)	71 (2, waitlist)	24	-	depressive mood, feeling anxious, stress, intrusive thoughts, avoidance, hyperarousal, positive thinking, self-efficacy
Breast cancer [30]	TM (T)	130 (2, usual care)	72	QoL ^A^, spiritual well-being, mental health	vitality
Cancer [31]	MBSR (M)	90 (2, waitlist)	7	depressive mood, feeling anxious, stress, anger, vigor, confusion, mood, habitual patterns, irritability	fatigue, peripheral symptoms
Diseases of the musculoskeletal system and connective tissues					
Rheumatoid arthritis [32]	Yoga-based mind-body intervention (Y)	72 (2, usual care)	8	depressive mood, inflammatory markers ^A^	-
Knee osteoarthritis [33]	Mantra (Y + T)	22 (2, music listening)	8	sleep, mood	QoL, stress, pain ^A^, osteoarthritis symptoms ^A^
Fibromyalgia [34]	Meditation awareness training (M)	148 (2, CBT)	8	depressive mood ^A^, feeling anxious ^A^, stress ^A^, sleep ^A^, pain ^A^, fibromyalgia symptoms ^A^, nonattachment, civic engagement	-
Chronic low back pain [35]	MBSR (M)	341 (3, CBT, usual care)	8	pain ^A^, disability ^A^, global impression	depressive mood, feeling anxious, QoL
Chronic low back pain [36]	Jyoti meditation (Y)	68 (2, exercise)	8	stress	depressive mood, feeling anxious, QoL, pain ^A^, disability
Fibromyalgia [37]	MBSR (M)	91 (2, usual care)	8	stress, sleep, fatigue, fibromyalgia symptoms	physical function, salivary cortisol
Chronic neck pain [38]	Jyoti meditation (Y)	89 (2, exercise)	8	pain ^A^	depressive mood, feeling anxious, QoL, stress
Chronic pain [39]	MBSR (M)	109 (2, waitlist)	8	depressive mood, feeling anxious, pain, vitality ^A^, general mental health, engagement	physical function, catastrophic thinking
Knee osteoarthritis [40]	Yoga (Y)	250 (2, physiotherapy exercise)	14	QoL	-
Chronic pain [41]	MBSR (M)	99 (2, multidisciplinary pain intervention)	8	vigorous activity	depressive mood, feeling anxious, QoL, pain ^A^, fatigue, anger, confusion
Fibromyalgia [42]	MBSR (M)	168 (3, nonspecific MBSR, waitlist)	8	-	depressive mood, feeling anxious, QoL ^A^, sleep, pain, mindfulness
Chronic low back pain [43]	Yoga (Y)	80 (2, exercise)	1	pain, flexibility	
Rheumatoid arthritis [44]	MM (M)	106 (3, CBT, education)	8	-	depressive mood ^A^, pain ^A^, affect ^A^, swelling, tenderness
Rheumatoid arthritis [45]	MBSR (M)	63 (2, waitlist)	8		depressive mood ^A^, rheumatoid arthritis symptoms ^A^, mental well-being, mindfulness
Diseases of the nervous system					
Parkinson’s disease [46]	Yoga (Y)	33 (2, proprioceptive training)	12	parkinsonism^A^	-
Headaches [47]	TM (T)	131 (3, hypnotherapy, muscle relaxation)	12	-	pain ^A^, depressive mood, feeling anxious, somatization
Multiple sclerosis [48]	Online MBSR (M)	121 (2, psychoeducation)	8	depressive mood, feeling anxious, QoL ^A^, sleep	fatigue
Migraines [49]	Spiritual meditation (T)	83 (4, internally & externally focused secular meditations, muscle relaxation)	4	pain	spirituality
Amyotrophic lateral sclerosis [50]	MBSR (M)	100 (2, usual care)	8	depressive mood, feeling anxious, QoL ^A^	mindfulness
Parkinson’s disease [51]	Mindfulness-based lifestyle (M)	57 (2, waitlist)	6	stress, mindfulness	depressive mood, feeling anxious, parkinsonism ^A^,
Multiple sclerosis [52]	Yoga (Y)	60 (2, no treatment)	12	-	QoL
Parkinson’s disease [53]	MBSR (M)	27 (2, usual care)	8	brain MRI (gray matter density) ^A^	-
Migraine [54]	Spiritual meditation (T)	83 (4, internally & externally focused secular meditations, muscle relaxation)	4	pain	depressive mood, feeling anxious, QoL, emotion, spirituality
Diseases of the circulatory system					
Heart disease [55]	VR meditation (other)	48 (2, eye mask or ear plugs)	1 day	sleep	-
Hypertension [56]	Yoga (Y)	75 (3, yoga without posture, waitlist)	12	blood pressure ^A^	depressive mood, feeling anxious, QoL, stress, heart rate
Hypertension [57]	MBSR (M)	101 (2, waitlist)	8	-	blood pressure ^A^
Congestive heart failure [58]	TM (T)	23 (2, education)	24	physical functions ^A^	depressive mood, QoL, stress
Coronary heart disease [59]	TM (T)	103 (2, education)	16	blood pressure, anger	depressive mood, feeling anxious, stress, blood lipid level, exercise, HRV
Hypertension [60]	TM (T)	150 (3, education, muscle relaxation)	48	blood pressure ^A^	-
Carotid atherosclerosis [61]	TM (T)	60 (2, education)	24	carotid intima-media thickness ^A^	blood pressure, blood lipid level, weight
Gynecological disorders					
Gynecological disease [62]	Mobile app Calm (M)	101 (2, usual care)	4	depressive mood, feeling anxious, stress ^A^, sleep	-
Chronic pelvic pain [63]	Mobile app-based MM (M)	90 (3, muscle relaxation, usual care)	8	-	pain ^A^
Dysmenorrhea [64]	Yoga (Y)	36 (2, no treatment)	12	stress, pain	-
Menopausal disorder [65]	Yoga (Y)	260 (2, no treatment)	18	QoL ^A^	-
Menopausal disorder [66]	Yoga (Y)	180 (2, no treatment)	12	menopausal symptoms ^A^	-
Menopausal disorder [67]	Yoga (Y)	120 (2, exercise)	8	stress, vasomotor symptoms, personality	psychological symptoms, somatic symptoms
Menopausal disorder [68]	Yoga (Y)	108 (2, exercise)	8	cognitive function ^A^	vasomotor symptoms ^A^
Metabolic disorders					
DM type 2 [69]	MBSR (M)	69 (3, muscle relaxation, education)	12	-	QoL, pain ^A^, fatigue
DM type 2 + amputation [70]	TM (T)	54 (2, diabetic care training)	4	amputee body image ^A^	-
Obesity [71]	MBSR (M)	194 (2, muscle relaxation)	22	-	sleep ^A^
DM type 2 [72]	MBSR (M)	56 (3, walking, education)	8	inflammatory response	stress^A^, blood glucose ^A^
DM type 2 [73]	Mindful eating intervention (M)	52 (2, self-management)	12	-	depressive mood, feeling anxious, DM symptoms ^A^, mindfulness
Metabolic syndrome [74]	Consciously resting meditation (Y + T)	68 (2, education)	12	stress, vascular functions ^A^	depressive mood, feeling anxious, hostility, anger, physical activity, metabolic & inflammatory markers
Diseases of otorhinolaryngology					
Glaucoma [75]	MM (M)	60 (2, medication)	3	QoL, IOP^A^	-
Glaucoma [76]	Breathing meditation (other)	60 (2, usual care)	6	-	QoL, IOP^A^, blood markers
Glaucoma [77]	MBSR (M)	90 (2, usual care)	3	QoL, IOP ^A^, blood markers	visual field
Tinnitus [78]	MM (M)	61 (2, relaxation)	15	tinnitus symptoms ^A^	depressive mood, feeling anxious, body temperature
Tinnitus [79]	MBCT (M)	75 (2, relaxation)	8	tinnitus symptoms ^A^, attention awareness	depressive mood, feeling anxious, stress ^A^, social adjustment
Diseases of the digestive system					
Foregut surgery [80]	VR meditation (other)	52 (2, usual care)	1 day	-	feeling anxious, pain, nausea
Functional gastrointestinal disorders [81]	Yoga (Y)	69 (2, usual care)	10	-	pain ^A^, well-being
Inflammatory bowel disease [82]	Mindfulness-based therapy (M)	66 (2, usual care)	16	-	stress, inflammatory bowel disease symptoms ^A^, positive thinking, avoidance, seeking advice, self-blame
Others					
COPD [83]	Breathing-based walking (other)	78 (2, usual care)	8	depressive mood, feeling anxious, COPD symptoms,	-
HIV [84]	Yoga (Y)	60 (2, waitlist)	8	depressive mood, feeling anxious, QoL ^A^, fatigue, well-being	-
Periodontitis [85]	Yoga (Y)	80 (2, usual care)	12	stress, periodontitis symptoms	-
Renal disease [86]	Telephone MBSR (M)	55 (2, telephone support)	8	depressive mood	feeling anxious ^A^, QoL, sleep, pain, fatigue
HIV [87]	Yoga (Y)	47 (2, usual care)	12	stress, positive affect ^A^, mental well-being ^A^, general health, social functions, cognitive functions	
Asthma [88]	Sahaja yoga (Y)	47 (2, relaxation + group discussion + CBT-like exercise)	16	feeling anxious, fatigue, asthma symptoms ^A^	depressive mood, QoL ^A^, anger, vigor, confusion

* The interventions were categorized in four groups (M: mindfulness-based; Y: yoga-based; T: transcendental; and other meditation). ^#^ This was in accordance with demographic feature of original article. ^A^ This is the primary outcome measurement of each RCT.CBT: cognitive behavior therapy; COPD: chronic obstructive pulmonary disease; DM: diabetes mellitus; HIV: human immunodeficiency virus; HRV: heart rate variability; IOP: intraocular pressure; MBCT: mindfulness-based cognitive therapy; MBSR: mindfulness-based stress reduction; MM: mindfulness meditation; MRI: magnetic resonance imaging; QoL: quality of life; TM: transcendental meditation; VR: virtual reality; YOCAS: yoga for cancer survivors.

**Table 3:** Summary of the RCTs for participants with mental disorders.

Disorder [Reference]	Kind of Meditation (Category *)	N. of Participants ^#^ (N. of Arms, Controls)	Period (Weeks)	Clinical Finding (Statistical)	
				Significant	Not Significant
Mood disorder					
Affective disorder [89]	MBCT (M)	104 (3, focused attention, open monitoring)	8	-	depressive mood ^A^, feeling anxious ^A^, stress ^A^
Depression [12]	Sahaj Samadhi (Y)	83 (2, usual care)	12	depressive mood, feeling anxious, global impression,	QoL, HRV ^A^, physical activity, side effects
Depression [90]	Laughter yoga (Y)	50 (2, usual care)	4	depressive mood, QoL	feeling anxious, stress
Depression [91]	Yoga-based lifestyle (Y)	178 (2, medication)	12	depressive mood ^A^	-
Depression [92]	Yoga-based lifestyle (Y)	58 (2, no treatment)	12	depressive mood ^A^, blood markers ^A^	-
Depression [93]	Yoga (Y)	26 (2, no treatment)	12	depressive mood, HRV	stress, heart rate
Depression [94]	MM (M)	74 (2, psychoeducation + regular rest)	2	depressive mood ^A^, mindfulness	-
Depression [95]	MBCT (M)	29 (2, usual care)	8	interoceptive awareness	depressive mood, pain
Depression [96]	MBCT (M)	43 (2, psychoeducation)	26	depressive mood, mindfulness, general mental health	feeling anxious
Depression [97]	MBCT (M)	254 (3, CPE, usual care)	8	-	depressive mood ^A^
Depression [98]	MBCT (M)	24 (2, no treatment)	8	sleep	depressive mood
Depression [99]	MBCT (M)	77 (2, waitlist)	8	depressive mood, rumination, mindfulness, positive thinking, negative thinking	
Depression [100]	MBCT (M)	21 (2, waitlist)	8	sleep	depressive mood
Post-traumatic stress disorder (PTSD)					
PTSD [101]	CBCT (other)	28 (2, Veteran. Calm)	10	PTSD symptoms ^A^	alcohol use
PTSD [102]	Mantra (Y + T)	173 (2, present-centered therapy)	8	sleep, PTSD symptoms ^A^	QoL, general health, anger, well-being, mindfulness
PTSD [13]	TM (T)	202 (3, prolonged exposure, education)	12	QoL, PTSD symptoms ^A^, general health, mood	
PTSD [103]	MM (M)	102 (4, MM + slow breathing, slow breathing, sitting quietly)	6	depressive mood, stress, sleep, PTSD symptoms ^A^, global impression, adherence, respiration rate	affect, mindfulness, credibility, EEG, ECG, HRV
Trauma with co-occurring disorder [104]	Tibetan meditation (other)	58 (2, usual care)	6	stress ^A^, trauma symptoms	-
PTSD [105]	Yoga (Y)	21 (2, waitlist)	1	feeling anxious, PTSD symptoms, arousal, respiration rate	depressive mood
PTSD [106]	Meditation-relaxation (other)	31 (2, exposure therapy)	4	-	PTSD symptoms ^A^, functional impairment, physical symptoms
Attention-deficit hyperactivity disorder (ADHD)					
ADHD + ODD [107]	MBSR (M)	50 (2, waitlist)	9	ADHD symptoms	-
ADHD [108]	MBCT (M)	120 (2, usual care)	8	ADHD symptoms ^A^, mindfulness, compassion, mental health	executive functions
ADHD [109]	Mindful awareness practice (M)	81 (2, psychoeducation)	8	mindfulness	depressive mood, QoL, ADHD symptoms ^A^
Anxiety					
Anxiety disorder [110]	Mindful exercise + psychoeducation (M)	91 (2, discussion forum)	8	depressive mood, feeling anxious ^A^, QoL, sleep	-
Anxiety disorder [111]	MBSR (M)	56 (2, exercise)	8	-	feeling anxious ^A^
Social anxiety disorder [112]	MBSR (M)	53 (2, CBT)	8	-	depressive mood, feeling anxious ^A^, QoL, global impression ^A^, social interaction ^A^, social phobia ^A^, interpersonal sensitivity
Sleep disorder					
Sleep disturbance [113]	Mobile app Calm (M)	263 (2, waitlist)	8	sleep, fatigue ^A^	-
Sleep disturbance [114]	Mindful awareness practice (M)	49 (2, education)	6	depressive mood, sleep ^A^, fatigue	feeling anxious, stress
Insomnia [115]	MBSR, MBTI (M)	54 (3, self-monitoring)	8	sleep	-
Substance misuse disorder					
Alcohol dependence [116]	MBRP (M)	112 (2, usual care)	8	-	stress, alcohol use ^A^, mindfulness
Alcohol and other drug use disorder [117]	MBRP (M)	168 (2, usual care)	8	-	alcohol or drug use, distress tolerance, mindfulness

* The interventions were categorized into four groups (M: mindfulness-based; Y: yoga-based; T: transcendental; and other meditation). ^#^ This was in accordance with demographic feature of original article. ^A^ This is the primary outcome measurement of each RCT. ADHD: attention-deficit hyperactivity disorder; CBCT: cognitively-based compassion training; CBT: cognitive behavior therapy; ECG: electrocardiography; EEG: electroencephalography; HRV: heart rate variability; MBCT: mindfulness-based cognitive therapy; MBSR: mindfulness-based stress reduction; MBRP: mindfulness-based relapse prevention; MBTI: mindfulness-based therapy for insomnia; MM: mindfulness meditation; MRI: magnetic resonance imaging; ODD: oppositional defiant disorder; PTSD: post-traumatic stress disorder; QoL: quality of life; TM: transcendental meditation.
